# Contributions of persisters and the Eagle effect to survival of *Yersinia pseudotuberculosis* under clinical antimicrobials

**DOI:** 10.1128/spectrum.00881-26

**Published:** 2026-07-23

**Authors:** Olivia Goode, Jilly Woodward, Urszula Łapińska, Sophia Zborowsky, Jemima Onime, Audrey Farbos, Paul O’Neill, Aaron Jeffries, Christopher H. Jenkins, Isobel H. Norville, Stefano Pagliara

**Affiliations:** 1 Living Systems Institute, University of Exeter, Exeter, United Kingdom; 2 School of Biosciences, Faculty of Health and Life Sciences, University of Exeter, Exeter, United Kingdom; 3 Dstl, Porton Down 512050 , Salisbury, United Kingdom; Universita degli Studi Roma Tre Dipartimento di Scienze, Rome, Italy

**Keywords:** persisters, antibiotic tolerance, eagle effect, antibiotics, hydrogen peroxide, catalase, gene expression, *Yersinia*

## Abstract

Microbes have evolved a variety of strategies to survive exposure to naturally occurring and synthetic antimicrobials. These strategies have been investigated extensively in model bacterial organisms, whereas less is known about under explored pathogenic bacteria such as bacteria within the *Yersinia* genus. In this study, we investigated the inhibitory effect and bactericidal activity of antibiotics from five different classes and of the disinfectant hydrogen peroxide against *Yersinia pseudotuberculosis*, the ancestral species from which *Yersinia pestis* and *Yersinia enterocolitica* have emerged. We found that *Y. pseudotuberculosis* is able to survive exposure to clinical antibiotics and disinfectants by employing a variety of strategies, with persisters and the Eagle effect playing a role in survival to quinolones, tolerance playing a role in survival to ceftriaxone and overexpression of catalases and peroxidases contributing to survival to hydrogen peroxide. Our findings suggest that future research should focus on informing new, effective ways to treat infections caused by *Yersinia* species.

**IMPORTANCE:**

Antimicrobial resistance is a complex phenomenon that constitutes one of the top global public health threats. Besides encoding genes that confer heritable resistance to antibiotic treatments, bacterial populations can also transiently survive antibiotic treatment, a phenomenon known as tolerance, or contain subpopulations, named persisters, that survive treatment while the majority of the population is killed by antibiotic treatment. Here, we investigate resistance, tolerance, and persistence of the bacterial pathogen *Yersinia pseudotuberculosis* to clinically relevant antibiotics and disinfectants. We show that *Y. pseudotuberculosis* populations contain persisters that survive treatment to two potent classes of antibiotics, the fluoroquinolones and cephalosporins, and to the widely used disinfectant hydrogen peroxide. Counterintuitively, increasing the concentration of fluoroquinolones employed decreases their activity against *Y. pseudotuberculosis*, a paradoxical effect known as the Eagle effect.

## INTRODUCTION

Infectious diseases are one of the leading causes of death worldwide, and antimicrobial resistance (AMR) poses a further challenge to global health with an estimated 5 million deaths associated with AMR every year (1). The misuse of antibiotics in human health and farming has accelerated the development of pathogens that are resistant to antibiotics (2–6), while the increased use of disinfectants since the coronavirus pandemic (7) raised concerns over the increase in biocide resistance (8).

Antibiotic resistance occurs at the genotypic level: bacterial populations either encode antibiotic resistance genes or acquire genetic changes during exposure to antibiotics (9–11) or via horizontal gene transfer (12). Bacterial populations can survive antibiotics also without encoding antibiotic resistance genes. In the case of tolerance, a bacterial population survives a transient exposure to antibiotics, even at concentrations that far exceed the minimum inhibitory concentration (MIC). In the case of persistence, antibiotic treatments that effectively kill the majority of the bacterial population do not kill subpopulations even within clonal cultures (13–15). Recent evidence suggests a significant increase in persisters in patients with relapsing infections (16) and a strong link between genetic resistance and persistence or tolerance (17–22), often collectively termed recalcitrance (23).

Recalcitrance to antibiotics has been investigated in depth *in vitro* (and to a lesser extent *in vivo* (23)) in bacterial model organisms, primarily *Escherichia coli* (24–27), *Salmonella enterica* (19, 28–30), *Mycobacterium tuberculosis* (31)*, Staphylococcus aureus* (32–35), and *Pseudomonas aeruginosa* (36–38). Several different processes have been identified to play a role in the recalcitrance of these organisms to antibiotics, including toxin-antitoxin systems (24, 39–41), stress response signaling molecules (42, 43), DNA damage repair systems and the SOS response (44–46), the formation of protein aggregates (47–50), and intracellular pH (15, 51).

In contrast, recalcitrance to antibiotics in other pathogens and to disinfectants has been investigated to a lesser extent (52). For example, *Yersinia* species are known to cause long-term infections, but little is known about recalcitrance to antibiotics and disinfectants in *Yersinia* (53, 54). *Yersinia* species are Gram-negative bacteria, with three species being pathogenic to humans: *Yersinia pestis*, *Yersinia enterocolitica*, and *Yersinia pseudotuberculosis*, the latter being the ancestral species from which the other two species emerged (55, 56). There are a variety of reservoirs in the environment which can serve as sources for *Yersinia* outbreaks including mammalian and avian hosts, soil, plants, insects, and contaminated water (57–63). Most cases occur in winter due to the cold tolerance of *Yersinia* which can survive and proliferate at low temperatures posing a potential food hazard (64). *Y. enterocolitica* and *Y. pseudotuberculosis* are enteric pathogens that are ingested through contaminated food, have a broad animal host range (56), and can colonize both the intestine and deep tissues retaining an extracellular lifestyle (65). Progression to systemic infection is relatively rare among patients, but if left untreated, patients succumb to systemic infection with an estimated mortality rate of 75%–100% (66).

Multidrug resistance has been reported for both *Y. pestis* and *Y. pseudotuberculosis,* and it has been linked to the acquisition of plasmids containing multiple resistance genes (67, 68). Moreover, around 10% of a *Y. pseudotuberculosis* population infecting mice spleen survived a single inhibitory dose of doxycycline (53). This subpopulation was primarily constituted of bacteria at the periphery of microcolonies that responded to host-produced nitric oxide and other stresses by expressing the detoxifying gene, *hmp* (69). This subpopulation may have persisted for days and resumed growth between 48 and 72 h posttreatment when the doxycycline concentration declined, ultimately leading to mice death (53). At a population level, the upregulation of the genes encoding the outer membrane protein OmpF and the outer membrane lipoprotein OsmB was recorded in response to inhibitory concentrations of doxycycline both *in vitro* and *in vivo*, together with the downregulation of the genes encoding the sulfur transfer complex subunit TusB and the cytotoxic necrotizing factor CnfY (54). OmpF seemed to perform the counterintuitive function of promoting diffusion of doxycycline out of the cell, whereas overexpression of *tusB* increased sensitivity to doxycycline (54). It has also been shown that the induction of *Yersinia* type-III secretion system leads to growth inhibition and reduced susceptibility to the antibiotic gentamicin *in vitro* and that *Y. pseudotuberculosis* bacteria surviving gentamicin in a mouse model also express low levels of *rpsJ*, which encodes the 30S ribosomal protein S10 (70).

These studies demonstrated that *Y. pseudotuberculosis* can display persistence or tolerance to gentamicin or doxycycline treatment. However, it remains unclear whether *Y. pseudotuberculosis* can display persistence or tolerance to high concentrations of a wider range of antibiotics and disinfectants that are routinely employed for antibiotic therapy or decontamination purposes. Here, we measure and compare susceptibility, persistence, and tolerance of *Y. pseudotuberculosis* to supra-inhibitory concentrations of antibiotics from five different classes recommended as first-line antibiotics by current guidelines from Centers for Disease Control and Prevention (71), and of hydrogen peroxide, which is used as a topical antiseptic and as an effective disinfectant (72–74). We further measure and compare the survival of the *Y. pseudotuberculosis* population after a 24 h exposure to supra-inhibitory concentrations of antibiotics or of hydrogen peroxide. We observed extensive survival of *Y. pseudotuberculosis* after 24 h exposure to high concentrations of hydrogen peroxide and, therefore, carried out genome-wide comparative transcriptome analysis of *Y. pseudotuberculosis* to identify changes in gene expression in response to the exposure to hydrogen peroxide. Under the *in vitro* conditions and strain tested in this study, *Y. pseudotuberculosis* is difficult to kill via either antibiotics or hydrogen peroxide and suggest that future research should focus on informing new, effective ways to treat *Y. pseudotuberculosis*.

## RESULTS

### Discrepancies between antimicrobials' minimum inhibitory concentrations and minimum bactericidal concentrations against *Yersinia pseudotuberculosis*

Using the broth microdilution assay (21), we investigated the susceptibility of *Y. pseudotuberculosis* to antimicrobial compounds, while the bacterium was in two distinct phases of growth, namely, exponential phase or stationary phase, i.e., after 6 h or 17 h of culture in Lysogeny broth (LB) medium at 37°C with shaking at 200 rpm, respectively. Indeed, *Y. pseudotuberculosis* remained in the lag phase of growth for the first 4 h of exposure to fresh LB, grew in exponential phase between 4 and 14 h, and remained in stationary phase between 14 and 24 h (Fig. S1) in accordance with previous reports (64, 75, 76).

We found that *Y. pseudotuberculosis* was equally susceptible when treated with antibiotics during exponential or stationary phase with the lowest minimum inhibitory concentration (MIC) value recorded for ceftriaxone (0.25 µg mL^−1^) and the highest MIC value recorded for gentamicin and chloramphenicol (4 µg mL^−1^). *Y. pseudotuberculosis* was instead slightly more susceptible to hydrogen peroxide when treated during exponential phase compared to stationary phase with MIC values of 1.25 mM and 2.5 mM, respectively (Table 1). Consistently, using colony-forming unit (CFU) assays (77), we found that the minimum bactericidal concentration (MBC) value of hydrogen peroxide was 8 mM against exponential phase *Y. pseudotuberculosis* and 16 mM against stationary phase *Y. pseudotuberculosis*. Similarly, the MBC value of ceftriaxone was slightly higher when *Y. pseudotuberculosis* was treated in stationary phase compared to exponential phase (8 µg mL^−1^ and 4 µg mL^−1^, respectively). The lowest MBC value was recorded for ciprofloxacin and levofloxacin (2 µg mL^−1^), whereas the highest MBC values were recorded for doxycycline and chloramphenicol (>64 µg mL^−1^) that are two bacteriostatic antibiotics (Table 1) although it has recently been suggested that doxycycline can also have bactericidal activity against *Y. pseudotuberculosis* (54). A relatively large discrepancy between the MIC and MBC values (>4-fold) was measured for doxycycline, gentamicin, ceftriaxone, and chloramphenicol, whereas a relatively small discrepancy (<4-fold) was measured for the two fluoroquinolones.

**TABLE 1:** *Y. pseudotuberculosis* susceptibility to antimicrobials^*a*^

	MIC values (µg mL^−1^/mM)		MBC values (µg mL^−1^/mM)	
	Exp. phase	Stat. phase	Exp. phase	Stat. phase
Doxycycline	1	1	>64	>64
Levofloxacin	1	1	2	2
Ciprofloxacin	0.5	0.5	2	2
Gentamicin	4	4	32	32
Ceftriaxone	0.25	0.25	4	8
Chloramphenicol	4	4	>64	>64
Hydrogen peroxide	1.25	2.5	8	16

^*a*^ MIC and MBC values measured for seven different antimicrobials against *Y. pseudotuberculosis* either in exponential or stationary phase. Values are reported in µg mL^−1^ for the six antibiotics tested and in mM for hydrogen peroxide. Values were obtained as the mean of measurements carried out in biological triplicates each consisting of technical triplicates.

### *Y. pseudotuberculosis* recalcitrance to antibiotics and hydrogen peroxide

Next, we set out to investigate both antimicrobial tolerance at the population level and the presence and levels of persisters to antimicrobials within clonal populations of *Y. pseudotuberculosis* in the stationary phase of growth by using supra-MIC antimicrobial concentrations and colony forming unit (CFU) assays.

**Fig 1:**
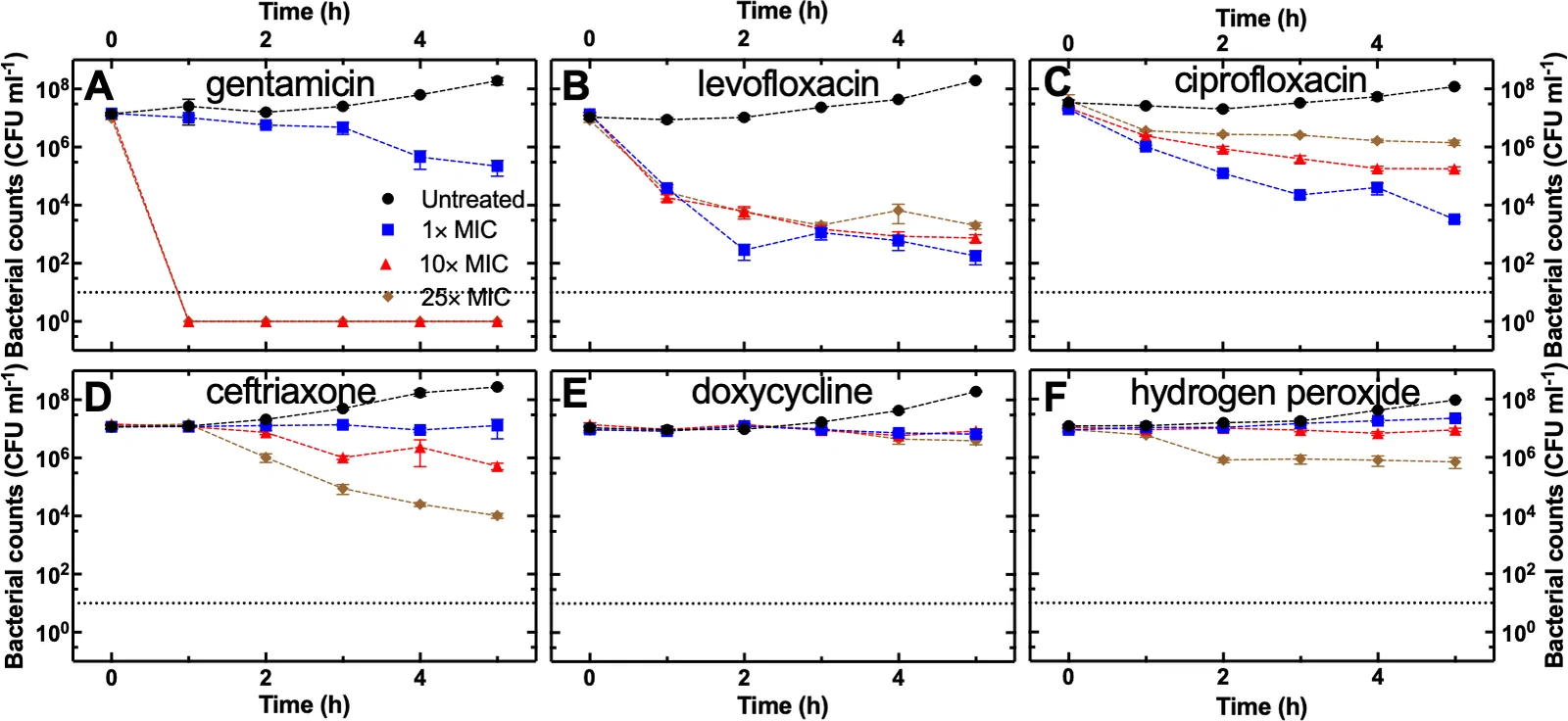
*Y*. *pseudotuberculosis* recalcitrance to antimicrobials**.** Time-dependent killing of *Y. pseudotuberculosis* by (**A**) gentamicin, (**B**) levofloxacin, (**C**) ciprofloxacin, (**D**) ceftriaxone, (**E**) doxycycline, or (**F**) hydrogen peroxide used at their MIC value (blue squares), 10× (red triangles) or 25× their MIC value (brown diamonds). Corresponding data for untreated control samples are reported with black circles in each graph. Symbols and error bars are the mean and standard error of biological triplicate each consisting of technical triplicate. The horizontal dotted lines indicate a limit of detection of 10 CFU mL^−1^.

We found that the aminoglycoside gentamicin displayed slow killing when used at 1× its MIC value (i.e., 4 µg mL^−1^) with a minimum duration of killing (MDK_99_) longer than 5 h (Fig. 1A), suggesting *Y. pseudotuberculosis* tolerance to gentamicin at the population level at this concentration. However, when this antibiotic was used at 10× or 25× its MIC value (i.e., 40 µg mL^−1^ thus slightly above gentamicin’s MBC value and 100 µg mL^−1^, respectively), both the MDK_99_ and the MDK_99.99_ values were less than 1 h (Table 2) with a complete killing of the *Y. pseudotuberculosis* population and no detectable survivors (Fig. 1A).

**TABLE 2:** *Y. pseudotuberculosis* tolerance to antimicrobials^*a*^

	MDK99 (h)			MDK99.99 (h)		
	1× MIC	10× MIC	25× MIC	1× MIC	10× MIC	25× MIC
Gentamicin	>5	<1	<1	>5	<1	<1
Levofloxacin	<1	<1	<1	2	4	>5
Ciprofloxacin	2	5	>5	>5	>5	>5
Ceftriaxone	>5	>5	3	>5	>5	>5
Doxycycline	>5	>5	>5	>5	>5	>5
Hydrogen peroxide	>5	>5	>5	>5	>5	>5

^*a*^ MDK_99_ and MDK_99.99_ values measured for six different antimicrobials against *Y. pseudotuberculosis*. Values were obtained as the mean of measurements carried out in biological triplicates each consisting of technical triplicates.

The third-generation fluoroquinolone levofloxacin displayed potent bactericidal activity with MDK_99_ and MDK_99.99_ values of less than 1 h and 2 h, respectively (Table 2), when this compound was used at 1× its MIC value (i.e., 1 µg mL^−1^, Fig. 1B), suggesting that *Y. pseudotuberculosis* is not tolerant to levofloxacin at the population level. After this initial rapid killing, the *Y. pseudotuberculosis* population did not further decrease from 2 h onward, the overall population dynamics following biphasic killing which indicates the presence of persisters (14). The *Y. pseudotuberculosis* persister fraction was 10^−5^ (i.e., one persister every a hundred thousand cells in the initial bacterial population) when levofloxacin was used at 1× its MIC value; surprisingly, increasing levofloxacin concentration to 10× or 25× its MIC value (i.e., 10 µg mL^−1^ and 25 µg mL^−1^, respectively) increased the MDK_99.99_ value to 4 h and more than 5 h, respectively (Table 2), also increasing the persister fraction to 5 × 10^−5^ and 10^−4^, respectively, after 5 h treatment (Fig. 1B). We found a similar trend also when we employed increasing concentrations of the second-generation fluoroquinolone ciprofloxacin. The MDK_99_ value increased from 2 h to 5 h and to more than 5 h (Table 2), and the *Y. pseudotuberculosis* persister fraction increased from 10^−4^ to 10^−2^ and to 10^−1^ when ciprofloxacin was used at 1×, 10×, or 25× its MIC value (i.e., 0.5 µg mL^−1^, 5 µg mL^−1^, and 12.5 µg mL^−1^, respectively, Fig. 1C). We also noted a slower more gradual killing of *Y. pseudotuberculosis* by ciprofloxacin compared to levofloxacin which is reflected in the larger MDK_99_ and MDK_99.99_ values (Table 2) and the less pronounced biphasic killing trends (compare Fig. 1B and C), suggesting *Y. pseudotuberculosis* tolerance to high concentrations of ciprofloxacin.

The third-generation cephalosporin ceftriaxone did not display bactericidal activity against *Y. pseudotuberculosis* when used at its MIC value (i.e., 0.25 µg mL^−1^) in accordance with the corresponding MBC data above. The MDK_99_ decreased from more than 5 h to 3 h (Table 2), and the *Y. pseudotuberculosis* survival fraction decreased from 4 × 10^−2^ to 10^−3^ when the ceftriaxone concentration was increased from 10× to 25× its MIC value (i.e., 2.5 µg mL^−1^ and 6.25 µg mL^−1^, respectively, Fig. 1D). Killing of *Y. pseudotuberculosis* was slow and gradual with MDK_99.99_ values larger than 5 h, suggesting tolerance to ceftriaxone at the population level, and it is also worth noting that no killing was recorded during the first hour of treatment with ceftriaxone (Fig. 1D). As expected, doxycycline being a bacteriostatic antibiotic did not display bactericidal activity against *Y. pseudotuberculosis* at any of the concentrations tested (Fig. 1E).

Finally, the disinfectant hydrogen peroxide displayed bactericidal activity against *Y. pseudotuberculosis* only when employed at 25× its MIC value (i.e., 62.5 mM) with a MDK_99_ longer than 5 h (Table 2) and a survival fraction of 10^−1^ (Fig. 1F), suggesting tolerance to hydrogen peroxide at the population level. Taken together, these data suggest that among the antimicrobials tested, fluoroquinolones provide the strongest bactericidal activity against *Y. pseudotuberculosis* at the lowest concentrations; however, the *Y. pseudotuberculosis* survival fraction depends strongly on the fluoroquinolone concentration which might be difficult to optimize *in vivo*.

### *Y. pseudotuberculosis* growth after prolonged exposure to antimicrobials

**Fig 2:**
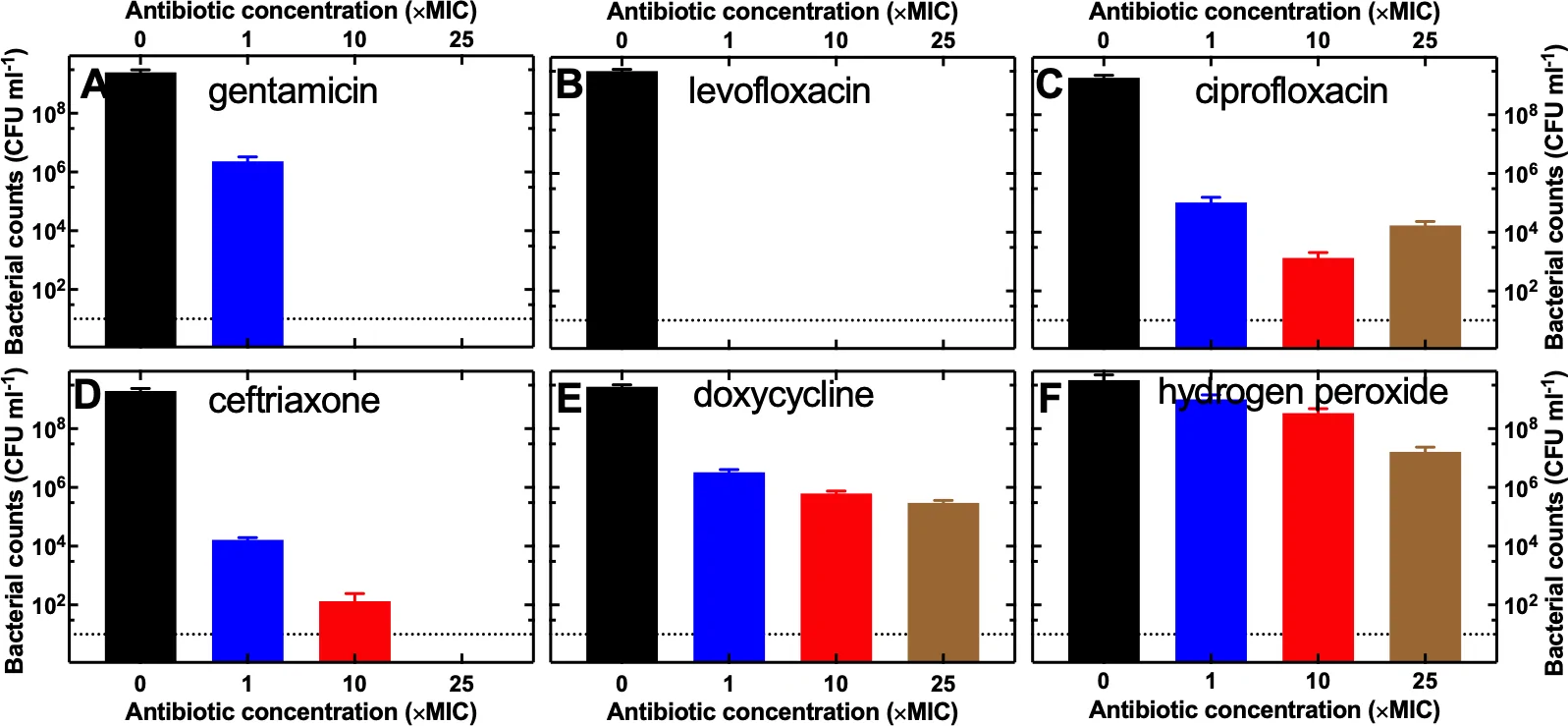
Y *pseudotuberculosis* growth after exposure to supra-MIC concentrations of antimicrobials. Counts of *Y. pseudotuberculosis* cells after 24 h exposure to (**A**) gentamicin, (**B**) levofloxacin, (**C**) ciprofloxacin, (**D**) ceftriaxone, (**E**) doxycycline, or (**F**) hydrogen peroxide used at 1×, 10×, or 25× their MIC value. Bars and error bars are the mean and standard error of biological triplicate each consisting of technical triplicate. Each set of experiments with a different antimicrobial included untreated control experiments without adding the antimicrobial, *Y. pseudotuberculosis* growth in these experiments is reported by the black bars (i.e., 0× the MIC value). The dotted lines indicate a limit of detection of 10 CFU mL^−1^.

Next, we carried out CFU assays after exposing *Y. pseudotuberculosis* to each of the antimicrobials above for 24 h. Consistent with the data above, we found that when used at its MIC value gentamicin caused a 3-log reduction in the number of culturable *Y. pseudotuberculosis* cells compared with untreated controls; whereas we did not detect any culturable *Y. pseudotuberculosis* cells when gentamicin was used at 10× or 25× its MIC value (Fig. 2A). Similarly, we did not detect any culturable *Y. pseudotuberculosis* cells after a 24 h exposure to levofloxacin at 1×, 10×, or 25× its MIC value (Fig. 2B). We measured a 4-log, 6-log and 5-log reduction in the number of culturable *Y. pseudotuberculosis* cells compared with untreated controls when ciprofloxacin was used at 1×, 10×, or 25× its MIC value, respectively (Fig. 2C). We measured a 5-log and 7-log reduction in the number of culturable *Y. pseudotuberculosis* cells compared with untreated controls when ceftriaxone was used at 1× or 10× its MIC, respectively, whereas we did not detect any culturable *Y. pseudotuberculosis* cells when ceftriaxone was used at 25× its MIC value (Fig. 2D). We measured a 3-log, 4-log, and 4-log reduction in the number of culturable *Y. pseudotuberculosis* cells compared with untreated controls when doxycycline was used at 1×, 10×, or 25× its MIC value (Fig. 2E). Prolonged exposure to hydrogen peroxide yielded a 0.5-log, 1-log, and 2-log reduction in the number of culturable *Y. pseudotuberculosis* cells compared with untreated controls when used at 1×, 10×, or 25× its MIC value, respectively (Fig. 2F). Together with the recalcitrance data above, these data suggest that levofloxacin is the most effective antimicrobial against *Y. pseudotuberculosis in vitro* and raise concerns about the use of other antimicrobials, particularly hydrogen peroxide, that is often employed as a disinfectant or a decontamination agent.

### *Y. pseudotuberculosis* is more susceptible to a second exposure to hydrogen peroxide

**Fig 3:**
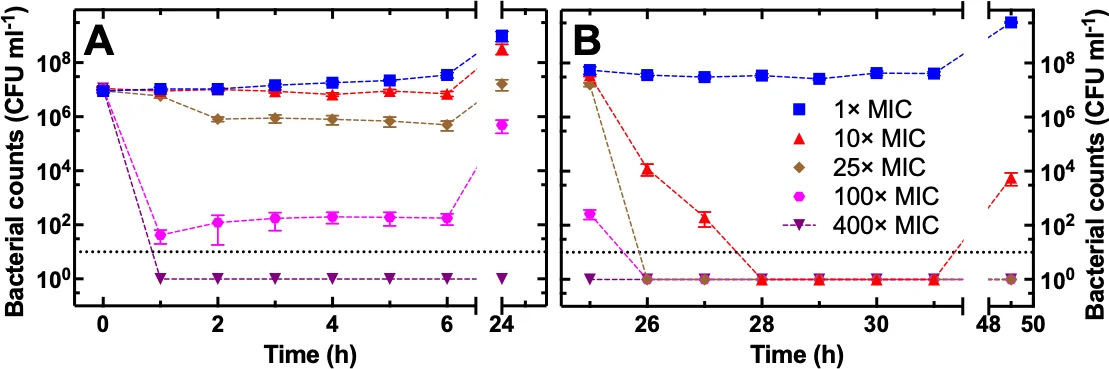
*Y. pseudotuberculosis* is more susceptible to a second exposure to hydrogen peroxide. Time-dependent killing of *Y. pseudotuberculosis* by (**A**) a first and (**B**) a second exposure to hydrogen peroxide used at its MIC value (blue squares), 10× (red triangles), 25× (brown diamonds), 100× (magenta hexagons), or 400× its MIC value (purple downward triangles). Symbols and error bars are the mean and standard error of biological triplicate each consisting of technical triplicate. The dotted lines indicate a limit of detection of 10 CFU mL^−1^.

We further investigated *Y. pseudotuberculosis* survival to hydrogen peroxide exposure and found a transition from *Y. pseudotuberculosis* population tolerance to subpopulation persistence when the concentration of hydrogen peroxide was increased from 25× to 100× its MIC value, with both MDK_99_ and MDK_99.99_ values decreasing from more than 5 h to less than 1 h (Fig. 3A). The *Y. pseudotuberculosis* persister fraction after exposure to hydrogen peroxide at 100× its MIC value was 10^−5^; this low number of cells was sufficient for *Y. pseudotuberculosis* regrowth to 0.5 million cells mL^−1^ after 24 h in the presence of hydrogen peroxide (Fig. 3A). *Y. pseudotuberculosis* was fully killed only after exposure to hydrogen peroxide used at 400× its MIC value (i.e., 1 M) with both MDK_99_ and MDK_99.99_ values of less than 1 h and no detectable persisters or regrowth after 24 h (Fig. 3A).

We then collected the *Y. pseudotuberculosis* cells that had survived different concentrations of hydrogen peroxide (i.e., 24 h time point in Fig. 3A), washed them in PBS to remove any residual hydrogen peroxide, resuspended them in 100 mL of LB, and exposed them a second time to the same concentration of hydrogen peroxide for another 24 h.

We found that *Y. pseudotuberculosis* was not killed by a second exposure to hydrogen peroxide at its MIC value similar to what we observed during the first exposure; however, when hydrogen peroxide was used at 10× its MIC value, *Y. pseudotuberculosis* regrowth after the second exposure was significantly lower compared to the first exposure (i.e., 10^3^ cells mL^−1^ vs 10^8^ cells mL^−1^, respectively, *P*-value < 0.0001 according to unpaired *t*-test), and we did not detect any persisters or regrowth during the second exposure to hydrogen peroxide at 25×, 100×, or 400× its MIC value (Fig. 3B).

Taken together, these data demonstrate that (i) *Y. pseudotuberculosis* survival and regrowth after the first exposure to hydrogen peroxide is not due to stable genetic mechanisms that can be passed on to the *Y. pseudotuberculosis* progeny; (ii) *Y. pseudotuberculosis* is more susceptible to a second exposure to hydrogen peroxide and, therefore, repeated applications of this antimicrobial agent can lead to *Y. pseudotuberculosis* complete killing provided that its concentration is sufficiently high, i.e., at least 25× its MIC value or 62.5 mM under our *in vitro* experimental conditions. In contrast, complete killing via a single exposure can be achieved only by using a very high concentration of hydrogen peroxide, i.e., 400× its MIC value or 1 M.

### Gene expression changes triggered by exposure to hydrogen peroxide

Next, we employed an unbiased approach to examine changes in gene expression by performing genome-wide comparative transcriptome analysis (78, 79) on *Y. pseudotuberculosis* after 6 h or 24 h of culture in LB or after 6 h or 24 h exposure to hydrogen peroxide at a concentration of 2.5 mM or 62.5 mM (i.e., 1× or 25× its MIC value). Samples were prepared for sequencing using our previous protocols for RNA-seq (78, 79) and the readings aligned to the *Y. pseudotuberculosis* genome (https://www.ncbi.nlm.nih.gov/nuccore/BX936398, see File S1 for RNA transcript counts for each replicate and File S2 for differential expression of individual genes in each condition compared to the corresponding untreated control experiments). Principal component analysis revealed major divergences across different culturing conditions and high within group reproducibility, i.e., transcriptome replicates from each condition clustered together (Fig. S2).

**Fig 4:**
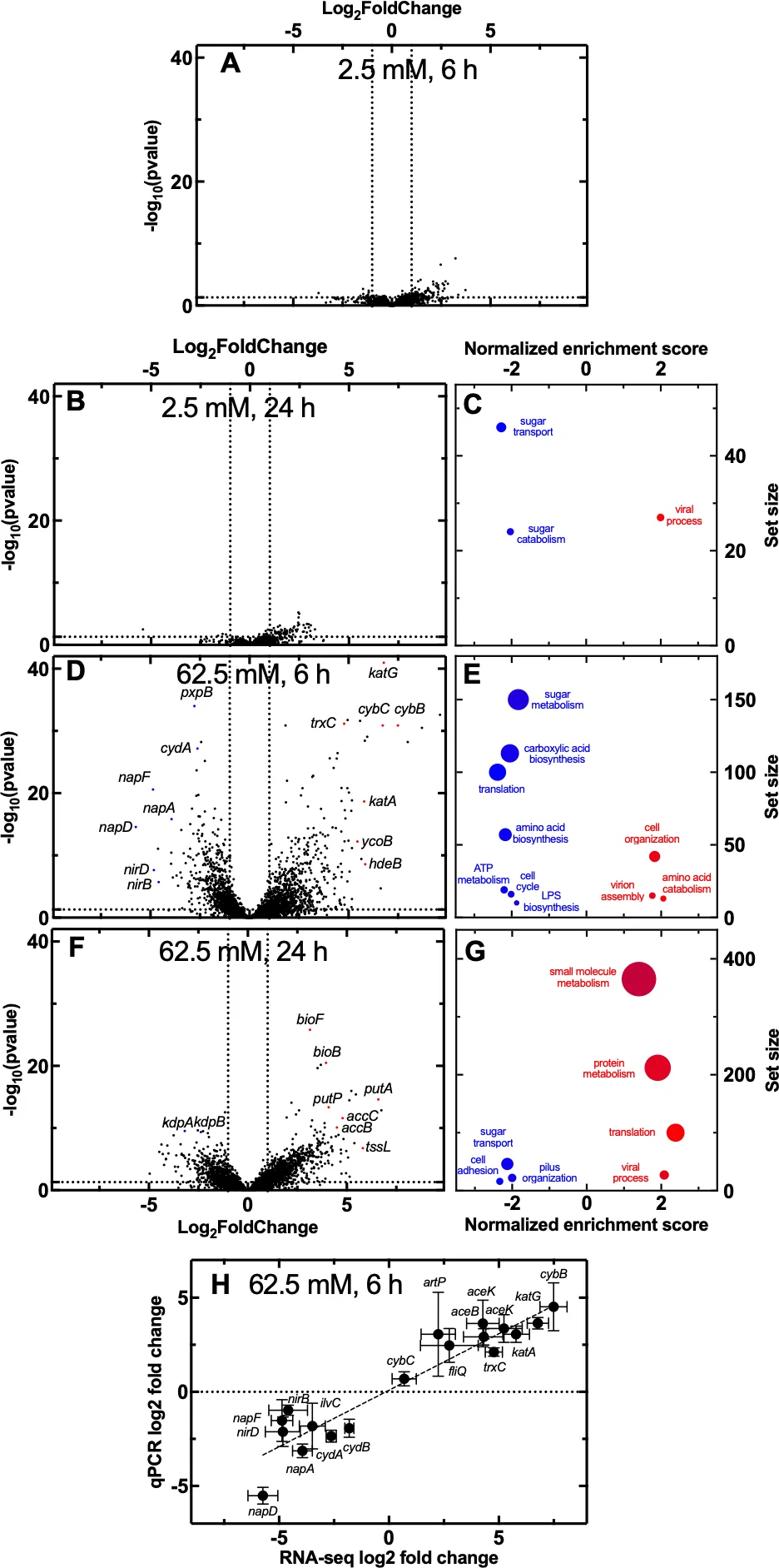
*Y****.*** *pseudotuberculosis* transcriptome rewiring in response to hydrogen peroxide. Volcano plot of whole-genome differential gene expression in *Y. pseudotuberculosis* after (**A**) 6 h or (**B**) 24 h exposure to 2.5 mM hydrogen peroxide or (**D**) 6 h or (**F**) 24 h exposure to 62.5 mM hydrogen peroxide compared to gene expression in corresponding untreated control experiments. Each dot represents the average log_2_ fold change in transcript reads of each gene and the corresponding adjusted *P*-value. Horizontal and vertical dotted lines indicate an adjusted *P*-value of 0.05 and a log_2_ fold change of 2, respectively. Strongly upregulated or downregulated genes of interest are highlighted in red and blue, respectively. Gene ontology enrichment analysis of differential gene expression in *Y. pseudotuberculosis* after (**C**) 6 h exposure to 62.5 mM hydrogen peroxide or (**E**) and (**G**) 24 h exposure to either 2.5 mM or 62.5 mM of hydrogen peroxide compared to untreated *Y. pseudotuberculosis* controls. Upregulated and downregulated processes are color coded red and blue, respectively, with their normalized enrichment score reported on the *x*-axis. The size of each circle represents the set size for the corresponding biological process with the numerical value reported on the *y*-axis. Normalized enrichment scores, set sizes, and adjusted *P*-values along with the lists of genes belonging to each biological process are reported in File S3 to S6. Each measurement was carried out in biological triplicate, and the values reported are the mean of these measurements (expressed as log_2_ fold change compared to untreated controls). Transcript read counts for the untreated controls as well as the error and *P*-value associated with each comparison are reported in File S2. RNA transcript counts for each replicate are reported in File S1. (**H**) Correlation between differential gene expression values obtained via RNA-seq (x-axis) and qPCR (*y*-axis) for 18 selected genes after 6 h exposure to 62.5 mM hydrogen peroxide compared to untreated control experiments.

Exposure to hydrogen peroxide for 6 h or 24 h at a concentration of 2.5 mM elicited a minor gene expression reprogramming in *Y. pseudotuberculosis* (Fig. 4A and B, respectively). Gene ontology enrichment analysis revealed that viral process was the only biological process upregulated after 24 h exposure to 2.5 mM hydrogen peroxide compared to untreated cultures, whereas carbohydrate transport and catabolism were downregulated (Fig. 4C and File S3 and S4).

Exposure to hydrogen peroxide for 6 h at a concentration of 62.5 mM elicited instead a major gene expression reprogramming in *Y. pseudotuberculosis* (Fig. 4D). The genes encoding the catalase-peroxidase and the catalase enzymes KatG and KatA were upregulated 6.8 and 5.8 log_2_ fold; *cybB* and *cybC*, encoding the cytochromes b561 and b562, were upregulated 7.5 and 6.7 log_2_ fold; the genes encoding the acid-activated periplasmic chaperone HdeB, the outer membrane protein YcoB and the reductase TrxC were upregulated 5.8, 4.8, and 5.5 log_2_ fold, respectively (Fig. 4D; File S2). On the other hand, the genes encoding the nitrate reductase complex NapAB, the cytochrome NapC, the chaperone NapD, and the ferredoxin-type protein NapF were downregulated 4.0, 2.4, 1.6, 5.7, and 4.9 log_2_ fold, respectively; the genes encoding the nitrite reductase subunit NirB and NirD and the nitrite transporter NirC were downregulated 4.6, 4.8, and 3.1 log_2_ fold, respectively; *cydA* and *cydB*, encoding the cytochrome ubiquinol oxidase subunits I and II, were downregulated 2.6 and 1.8 log_2_ fold, respectively; the gene encoding the 5-oxoprolinase subunit PxpB was downregulated 2.8-fold (Fig. 4D; File S2). Furthermore, gene ontology enrichment analysis revealed that cell organization, amino acid catabolism, and virion assembly were upregulated after 6 h exposure to 62.5 mM hydrogen peroxide compared to untreated cultures, whereas translation, carbohydrate metabolism, amino acid, carboxylic acid and lipopolysaccharide biosynthesis, ATP metabolism, and cell cycle processes were downregulated (Fig. 4E; File S5).

After 24 h exposure to hydrogen peroxide at a concentration of 62.5 mM, a different set of genes was strongly differentially regulated: the genes encoding the proline dehydrogenase PutA and the sodium/proline symporter PutP, the type IV secretion system protein TssL, the acetyl-CoA carboxylase subunits AccB and AccC, and the biotin synthases BioB and BioF were upregulated 6.6, 4.1, 5.8, 4.6, 4.8, 4.0, and 3.2 log_2_ fold, respectively; the genes encoding the potassium-transporting ATPase subunits KdpA and KdpB were downregulated 3.2 and 2.4 log_2_ fold, respectively (Fig. 4F). Interestingly, gene ontology enrichment analysis revealed that translation was in this case upregulated alongside viral process, small molecule, and protein metabolism, whereas carbohydrate transport, cell adhesion, and pilus organization were downregulated after 24 h exposure to 62.5 mM hydrogen peroxide compared to untreated cultures (Fig. 4G; File S6).

These RNA-seq data were further validated by quantitative real-time PCR for 18 genes (including strongly upregulated genes *katG*, *katA*, *cybB*, *cybC* genes and strongly downregulated genes *cydA*, *cydB*, *nirB*, *nirD*), showing a strong correlation with the transcriptome data (Pearson correlation coefficient of 0.94 and a *P*-value < 0.0001, Fig. 4H) after 6 h exposure to 62.5 mM hydrogen peroxide.

## DISCUSSION

*Y. pseudotuberculosis* is a model organism for *Y. pestis,* which is the causative agent of plague and is on the WHO list of high risk organisms that could cause the next pandemic (80). Indeed, Madagascar witnessed a major plague outbreak in 2017 with at least one *Y*. *pestis* strain displaying antimicrobial resistance (81). Here, we set out to investigate persistence and tolerance to antibiotics and disinfectants in *Y. pseudotuberculosis* considering that recent evidence suggests a significant increase in persisters in patients with relapsing infections (16) and a strong link between genetic resistance and persistence or tolerance in other pathogenic bacteria (17–22).

The minimum inhibitory concentration values we recorded for doxycycline, levofloxacin, ciprofloxacin, and gentamicin against *Y. pseudotuberculosis* are in line with the range of values in previous reports (53, 54, 82–84). These MIC values are above the breakpoint values recommended by EUCAST for the use of these antibiotics against Enterobacterales. Moreover, although relatively high, these antibiotic concentrations are not sufficient to kill *Y. pseudotuberculosis*, the minimum bactericidal concentration values being between 2-fold and 64-fold larger than the corresponding minimum inhibitory concentration values measured for each antibiotic. The scarce efficacy of these clinical antibiotics is further exacerbated by *Y. pseudotuberculosis* also displaying either persistence or tolerance to levofloxacin, ciprofloxacin, and ceftriaxone which leads to the regrowth of *Y. pseudotuberculosis* after 24 h exposure to these antimicrobials. Only the use of gentamicin at a minimal concentration of 40 µg mL^−1^ or levofloxacin at 1 µg mL^−1^ led to *Y. pseudotuberculosis* complete killing after 24 h treatment. However, it should be noted that we used only one *Y*. *pseudotuberculosis* strain and that we tested only *in vitro* conditions.

These data demonstrate that *Y. pseudotuberculosis* is difficult to treat with antibiotics under *in vitro* conditions and are in line with recent reports demonstrating that *Y. pseudotuberculosis* can survive doxycycline treatment within host mouse tissues (53, 54). The strong efficacy we recorded for levofloxacin *in vitro* is also in line with previous evidence of good efficacy of levofloxacin and poor efficacy of doxycycline against pneumonic plague in a nonhuman primate model (85).

Extensive investigations have been carried out on persistence and tolerance to antibiotics, primarily using bacterial model organisms, whereas little is known about persistence and tolerance in *Yersinia* species. Persisters to meropenem and to multiple antibiotics, including ciprofloxacin, were found in attenuated *Y. pestis* (86) and in *Y. ruckerii* (87), respectively, whereas a subpopulation of *Y. enterocolitica* cells was able to survive whole blood complement (88). The new data we obtained here for *Y. pseudotuberculosis* are in line with some previous reports of persisters in different species. For example, a biphasic kill curve characteristic of persister presence was recorded when levofloxacin was used at a concentration of 5 µg mL^−1^ against *E. coli* (46), in line with our data showing *Y. pseudotuberculosis* persisters to levofloxacin at concentrations of 10 µg mL^−1^. *Y. ruckeri* persisters to ciprofloxacin were observed when this antibiotic was used at 10 µg mL^−1^ (87), and we also found *Y. pseudotuberculosis* persisters when using ciprofloxacin at 12.5 µg mL^−1^.

While for gentamicin, ceftriaxone, and hydrogen peroxide bactericidal efficacy increased with antibiotic concentration as expected (89–91), the opposite was true for the two fluoroquinolones tested, ciprofloxacin and levofloxacin. These paradoxical observations resemble the Eagle effect that was first identified in 1948 when it was observed that there was an optimal penicillin concentration beyond which the efficacy of this antibiotic in killing several bacterial species sharply decreased (92). Investigations of the Eagle effect have been carried out in Gram-positive and Gram-negative bacteria, mycobacteria, and fungi using a diverse range of antimicrobials; however, its underpinning mechanisms are not well understood, partly due to the fact that the Eagle effect seems to be both antibiotic- and strain-dependent (93).

The Eagle effect has been observed for ciprofloxacin against *E. coli* (94), *Mycobacterium smegmatis* (95), *S. aureus*, *S. pneumoniae,* and *P. aeruginosa* (96), whereas here, we report for the first time that the efficacy of levofloxacin and ciprofloxacin against a *Y. pseudotuberculosis* strain decreases with the concentration of these drugs. Specifically, our data showing that ciprofloxacin efficacy against *Y. pseudotuberculosis* decreases at concentrations that are from 10× to 25× the MIC value (i.e., 5–12.5 µg mL^−1^) are in line with previous data: ciprofloxacin efficacy against *S. aureus*, *S. pneumoniae,* and *P. aeruginosa* decreased at concentrations above 35× the MIC value (i.e., 14 µg mL^−1^) when bactericidal activity was measured in serum ultrafiltrate (96). However, it is worth reiterating that the Eagle effect appears to be strain-dependent: for example, we and others have not previously observed the Eagle effect for ciprofloxacin against *E. coli* (21) or *S. aureus* (91). Postulated mechanisms behind the Eagle effect induced by quinolone antibiotics are related to suppression of reactive oxygen species accumulation at high quinolone concentrations (97). Future investigations should test whether this mechanism plays a role in the Eagle effect we observed when using fluoroquinolones against *Y. pseudotuberculosis*.

Considering that animal models support that the Eagle effect observed *in vitro* translates to *in vivo* consequences, including in human patients (93), further investigation of this effect in *Yersinia* species within host mouse tissues (53, 54) could lead to clinical benefits, where low antibiotic exposures may expedite clearance of infection. Moreover, recent evidence suggests an increase in ceftriaxone persisters in relapsed *E. coli* infection (16) and that *S. typhimurium* persisters into the gut promote resistance-plasmid transfer between other gut Enterobacteriaceae (19). Therefore, it would be valuable to investigate whether high persister isolates are also present in relapsed infecting strains of *Yersinia* species and the minimum infectious dose of persisters that causes an infection to relapse *in vivo*. It would also be valuable to investigate the mechanisms underpinning *Yersinia* persisters to antibiotics considering that mechanistic insights are so far limited to doxycycline only (53, 54).

We found that *Y. pseudotuberculosis* was difficult to eradicate also by using hydrogen peroxide, which is employed as a topical antiseptic and as an effective disinfectant (72–74). Previous reports suggested that hydrogen peroxide can be either bacteriostatic or bactericidal depending on the concentration employed (76, 98, 99). In line with these observations, we show that hydrogen peroxide starts to display some bactericidal activity against *Y. pseudotuberculosis* when used at concentrations higher than 10× its MIC value. We found a biphasic kill curve characteristic of persister presence when we used hydrogen peroxide at 100× its MIC value, whereas recent reports identified *E. coli* persistence to benzalkonium chloride used at 3× its MIC value, but no persisters to hydrogen peroxide (100, 101). Complete killing of *Y. pseudotuberculosis* was obtained only when hydrogen peroxide was used at 400× its MIC value, that is 1 M concentration or via two 24 h exposures to hydrogen peroxide at 25× its MIC value, suggesting a possible alternative to the use of highly concentrated hydrogen peroxide solutions.

Bacteria employ catalases and peroxidases to convert hydrogen peroxide to non-toxic oxygen and water, thus reducing intracellular concentrations of hydrogen peroxide (102). Catalase deletion mutants of planktonic *Salmonella enterica* serovar Typhimurium have displayed decreased MIC values and reduced tolerance in biofilms to hydrogen peroxide (103). Catalase mutants in *E. coli* accumulate enough hydrogen peroxide to cause substantial damage to DNA and display growth and viability defects (104). Furthermore, RNA transcriptome analysis of *Y. pseudotuberculosis* after 5 min of inhibitory concentrations of hydrogen peroxide revealed enrichment of genes involved in oxidative stress response, including OxyR regulon members and downregulation of metabolic enzymes (99). The activation of OxyR in response to hydrogen peroxide was also identified in *E. coli* after hydrogen peroxide exposure (105).

Using RNA sequencing and qPCR, we found that *Y. pseudotuberculosis* strongly upregulates the genes encoding the catalase-peroxidase and the catalase enzymes KatG and KatA after 6 h exposure to a supra-inhibitory and bactericidal concentration of hydrogen peroxide. More broadly, amino acid catabolism was upregulated at this time point, whereas amino acid biosynthesis, translation, and ATP metabolism were downregulated. *katA* and *katG* were then downregulated after 24 h incubation in hydrogen peroxide, suggesting that by using these catalases *Y. pseudotuberculosis* had decreased both the intracellular and extracellular concentration of hydrogen peroxide by this time point allowing growth to restart. Interestingly, the genes and processes above were not upregulated after exposure to an inhibitory but not bactericidal concentration of hydrogen peroxide, suggesting that *Y. pseudotuberculosis* can tolerate this concentration of hydrogen peroxide by being in a non-growing state and with a minor transcriptome rewiring.

Future investigations should directly measure how catalases and peroxidases, produced by *Yersinia* species, impact the extracellular concentration of hydrogen peroxide, whereas catalase negative mutants could be used to determine the impact of these enzymes on the MIC and MBC values of hydrogen peroxide against *Yersinia*. It would also be important to investigate cross tolerance and persistence by exposing *Yersinia* to both hydrogen peroxide and antibiotics, considering that exposure to hydrogen peroxide has been reported to increase the percentage of *E. coli* persisters to antibiotics (100, 106, 107).

In summary, we show that *Y. pseudotuberculosis* is able to survive treatment with clinical antibiotics and disinfectants by employing a variety of strategies, with persisters playing a role in survival to quinolones, tolerance playing a role in survival to ceftriaxone and catalases contributing to survival to hydrogen peroxide.

## MATERIALS AND METHODS

### Strains and culture conditions

All chemicals were purchased from Merck or Fischer Scientific unless otherwise stated. *Yersinia pseudotuberculosis* strain IP32953 (genome available at https://www.ncbi.nlm.nih.gov/nuccore/BX936398) was kindly provided by the Defence Science and Technology Laboratory. *Y. pseudotuberculosis* was streaked on LB medium agar plates (15 g/L agar, 10 g/L tryptone, 5 g/L yeast extract, and 10 g/L NaCl; Melford) from cryostock. Overnight liquid cultures were prepared by picking a single colony from a streak plate and grown in 200 mL of LB medium and incubated for 17 h at 37°C with shaking at 200 rpm.

### Measurement of minimum inhibitory concentration and minimum bactericidal concentration values of antimicrobials against *Y. pseudotuberculosis*

MIC assays were carried out using the 96-well plate microdilution method as previously described (108, 109). Briefly, *Y. pseudotuberculosis* was grown in LB for 17 h at 37°C while shaking at 200 rpm. To establish the MIC of antimicrobials against *Y. pseudotuberculosis* in exponential phase, the overnight culture was diluted to an OD_600_ of 0.01 and incubated at 37°C for 6 h either in cation-adjusted Muller-Hinton broth or in LB medium before their addition to the 96-well plate containing antimicrobials at a final bacterial OD_600_ of 0.01. To determine the MIC of antimicrobials against *Y. pseudotuberculosis* in stationary phase, the overnight culture was diluted either in cation-adjusted Muller-Hinton broth or in LB medium and immediately added to the 96-well plate containing antimicrobials at a final bacterial OD_600_ of 0.01. The dilution step was carried out to obtain a starting inoculation at roughly 1 × 10^6^ CFU/mL. The 96-well plates were incubated for 24 h at 37°C while shaking at 200 rpm. After 24 h, the OD_600_ of each individual well was measured and analyzed with a plate reader as previously described (110). The average OD_600_ value measured in three wells containing either cation-adjusted Muller-Hinton broth or LB medium only was subtracted from the OD_600_ value in each of all the other wells on the plate to obtain a background-subtracted OD_600_ measurement. The MIC value for each antimicrobial in each plate was determined as the minimal antimicrobial concentration for which the associated OD_600_ value was smaller than 10% of the value obtained as an average OD_600_ of untreated wells, i.e., containing *Y. pseudotuberculosis* in LB. All measurements were performed in biological and technical triplicates. Measurements carried out in cation-adjusted Muller-Hinton broth or LB medium yielded the same MIC values.

To measure the MBC value of each antimicrobial against *Y. pseudotuberculosis*, the MIC assays were repeated and, after 24 h, aliquots from wells with antimicrobial concentrations equal to or above the MIC value were resuspended in PBS and plated on LB agar plates. Such plates were incubated for 24 h at 37°C, and the MBC was defined as the lowest antimicrobial concentration required to give a 3 log_10_, i.e., 99.9% killing, reduction in surviving bacteria compared to the initial inoculum (93).

### Time-kill assays

Time-kill assays were performed as previously reported (47, 78). Briefly, an overnight culture of *Y. pseudotuberculosis* was prepared as described above, diluted in 100 mL LB to an OD_600_ of 0.03 (equivalent to around 10^7^ bacteria mL^−1^) and either exposed to each antimicrobial at a concentration of 1×, 10×, or 25× its MIC value or incubated in LB only as an untreated control sample. All cultures were incubated at 37°C at 200 rpm for 24 h. Triplicate aliquots were withdrawn from each culture at hourly time points for the first 5 h and after 24 h. Each aliquot was washed in PBS and serially diluted, 10 µL of each dilution was spotted on LB agar plates and incubated at 37°C, colony-forming units (CFU) were counted on each plate after 24 h incubation, with a limit of detection of 10 CFU mL^−1^ since we also spotted 100 µL of undiluted samples. For samples in which we did not find any CFUs, we assigned an indicative numerical value of 1, i.e., lower than the limit of detection, but still compatible with the log_10_ scale representation used in the graphs. Each experiment was performed in biological triplicate.

The minimum duration of killing 99% (MDK_99_, 2 log_10_ reduction) or 99.99% (MDK_99.99_, 4 log_10_ reduction) of the initial inoculum was measured for each antimicrobial against *Y. pseudotuberculosis* in line with previous definitions (13). Persisters were identified via the observation of biphasic killing with a sharp initial reduction in surviving bacteria followed by a slower reduction and a plateau in the number of survival bacteria; the persister fraction was defined as the ratio between the number of surviving bacteria when this plateau was reached over the number of bacteria in the initial inoculum (15).

For hydrogen peroxide, a second exposure was carried out using cultures already exposed to 2.5, 25, 62.5, and 250 mM hydrogen peroxide for 24 h. Cultures were resuspended to an OD_600_ of 0.03 with the exception of cultures previously exposed to 250 mM hydrogen peroxide in which the whole culture was centrifuged and resuspended in LB to capture the highest number of cells present. The cultures were resuspended in LB and the same concentration of hydrogen peroxide to which they had been previously exposed. Colony-forming unit assays were carried out for each culture as described above.

### Transcriptomic analysis

RNA extraction, sequencing, and analysis were performed on biological triplicate of each condition following previously reported protocols (35, 111). Briefly, overnight cultures of *Y. pseudotuberculosis* were diluted in 100 mL LB to an OD_600_ of 0.03 with the addition of 2.5 or 62.5 mM of hydrogen peroxide or without addition of hydrogen peroxide. Three 100 µL aliquots were withdrawn from each culture either after 6 h or 24 h transferred into an RNase-free tube and centrifuged at 4,000 rpm for 15 min. Two hundred microliters of RNA-protect bacteria reagent was added, vortexed for 5 s, and then incubated for 5 min at room temperature. Each sample was then centrifuged at 5,000 *g* for 10 min, and the supernatant was removed. Extractions were performed using RNeasy minikit (Qiagen) by following the manufacturer’s instructions. DNA removal during extraction was carried out by using RNase-Free DNase I kit (Qiagen). RNA concentration and quality were measured using a Qubit 4.0 fluorometer (Invitrogen) and 4200 TapeStation (Agilent), respectively, and only samples with an RNA integrity number larger than 7.5 were taken forward and sequenced. Transcript abundance was quantified using Salmon for each gene in all samples. Subsequent differential analysis was performed using DEseq2 in R software to quantify the log_2_ fold change in transcript reads for each gene (112). Gene set enrichment analysis was performed using the clusterProfiler package (version 4.10.0) for R as previously described (113). Enrichment in terms belonging to the “Biological Process” ontology was calculated relative to the set of all genes quantified in the experiment, via an Enrichment Score, utilizing a weighted Kolmogorov-Smirnov(KS)-like statistic (114). *P*-values were adjusted for false discovery by using the method of Benjamini and Hochberg. The lists of significantly enriched terms were simplified to remove redundant terms, as assessed via their semantic similarity to other enriched terms, using clusterProfiler’s simplify function.

### Quantitative real-time PCR

Overnight cultures were grown in LB at 37°C with shaking at 200 rpm and subsequently diluted to an OD₆₀₀ of 0.03 (1:200 dilution) in fresh medium. Three independent biological replicates were prepared for each condition. Each biological replicate was divided into two, with one receiving hydrogen peroxide to a final concentration of 62.5 mM, while the other served as an untreated control. Cultures were incubated at 37°C with shaking at 200 rpm for 6 h.

Following the 6 h treatment period, cultures were collected by centrifugation at 4,000 rpm for 15 min. Cell pellets containing around 10⁹ cells were resuspended in TE buffer (Invitrogen) containing lysozyme (Sigma-Aldrich) at a final concentration of 1 mg mL^−1^ and incubated at 37°C for 15 min to facilitate cell lysis.

Total RNA was purified using the RNeasy Midi Kit (Qiagen) according to the manufacturer’s protocol. Genomic DNA contamination was removed during purification using the kit’s on-column DNase digestion step. For reverse transcription, 400 ng of total RNA was converted to cDNA using the High-Capacity cDNA Reverse Transcription Kit (Applied Biosystems) with random hexamer primers. Reverse transcription reactions were performed in a final volume of 20 µL and consisted of primer annealing at 25°C for 10 min, cDNA synthesis at 37°C for 120 min, and enzyme inactivation at 85°C for 5 min. The resulting cDNA was diluted fivefold in 10 mM Tris-HCl (pH 8.0) and stored at −70°C until further analysis.

Quantitative PCR was carried out using PowerUp SYBR Green Master Mix (Applied Biosystems) in 20 µL reaction volumes containing 1 × master mix, 600 nM each of the forward and reverse primers (Table S1), and 10 ng of cDNA template. Amplification was performed on a QuantStudio 1 Real-Time PCR System (Thermo Fisher Scientific). Thermal cycling conditions consisted of an initial UDG activation step at 50°C for 2 min, followed by polymerase activation at 95°C for 2 min, and 35 cycles of denaturation at 95°C for 1 s and annealing/extension at 60°C for 30 s.

To confirm amplification specificity, a melt curve analysis was performed following amplification by increasing the temperature from 60°C to 95°C at a rate of 0.1°C s⁻¹ with continuous fluorescence acquisition. Threshold cycle (Ct) values were determined using Design & Analysis (DA2) software version 2.6.0 (Thermo Fisher Scientific).

Relative gene expression levels were calculated using the comparative Ct (ΔΔCt) method. Expression values were normalized against the housekeeping gene *rbfA* to account for differences in RNA input and reverse transcription efficiency between samples. Gene expression changes are reported as log₂ fold changes relative to the corresponding untreated controls.

### Statistical analysis

Points and error bars displayed in all graphs represent the mean and standard error of the mean. Biological triplicate experiments were conducted in all instances. All graphs were generated using GraphPad Prism 9.

## Supplementary Material

Reviewer comments

## Data Availability

All data generated or analyzed during this study are included in this published article and its supplemental files. RNA-seq data have been deposited in the NCBI Sequence Read Archive under BioProject accession PRJNA1480040. Associated SRA run accessions are SRR39232933–SRR39232951.

## SUPPLEMENTAL MATERIAL

Supplemental legends spectrum.00881-26-s0001.docx Descriptive legends for Fig. S1 and S2.

Fig. S1 spectrum.00881-26-s0002.tiff Temporal dependence of bacterial counts for *Y. pseudotuberculosis* growing in LB medium at 37°C.

Fig. S2 spectrum.00881-26-s0003.tiff Principal component analysis of replicate transcriptomes of *Y. pseudotuberculosis* after 6 h or 24 h of incubation in LB or exposure to 2.5 mM or 62.5 mM hydrogen peroxide.

File S1 spectrum.00881-26-s0004.xlsx Transcript counts for each aligned *Y. pseudotuberculosis* gene and for each replicate of each experimental condition.

File S2 spectrum.00881-26-s0005.xlsx Differential expression of *Y. pseudotuberculosis* genes after 6 h or 24 h of exposure to 2.5 mM or 62.5 mM hydrogen peroxide compared to 6 h or 24 h incubation in LB.

File S3 spectrum.00881-26-s0006.xlsx Gene ontology enrichment analysis of differential gene expression in *Y. pseudotuberculosis* after 6 h exposure to 2.5 mM hydrogen peroxide compared to 6 h or 24 h incubation in LB.

File S4 spectrum.00881-26-s0007.xlsx Gene ontology enrichment analysis of differential gene expression in *Y. pseudotuberculosis* after 24 h exposure to 2.5 mM hydrogen peroxide compared to 6 h or 24 h incubation in LB.

File S5 spectrum.00881-26-s0008.xlsx Gene ontology enrichment analysis of differential gene expression in *Y. pseudotuberculosis* after 6 h exposure to 62.5 mM hydrogen peroxide compared to 6 h or 24 h incubation in LB.

File S6 spectrum.00881-26-s0009.xlsx Gene ontology enrichment analysis of differential gene expression in *Y. pseudotuberculosis* after 24 h exposure to 62.5 mM hydrogen peroxide compared to 6 h or 24 h incubation in LB.

Table S1 spectrum.00881-26-s0010.xlsx List of primers employed in the qPCR analysis.

OPEN PEER REVIEW reviewer-comments.pdf An accounting of the reviewer comments and feedback.

ASM does not own the copyrights to Supplemental Material that may be linked to, or accessed through, an article. The authors have granted ASM a non-exclusive, world-wide license to publish the Supplemental Material files. Please contact the corresponding author directly for reuse.
