# Supplementary material for: Contributions of persisters and the Eagle effect to survival of Yersinia pseudotuberculosis under clinical antimicrobials
Source: Microbiol Spectr. 2026 Jul 23;14(9):e00881-26. doi: 10.1128/spectrum.00881-26 (PMC13532240; doi:10.1128/spectrum.00881-26)
Supplement: Supplementary file 1 [file reviewer-comments.pdf]

AMERICAN SOCIETY FOR MICROBIOLOGY | Microbiology Spectrum

Contributions of persisters and the Eagle effect to survival of *Yersinia pseudotuberculosis* under clinical antimicrobials

Olivia Goode, Jilly Woodward, Urszula Lapinska, Sophia Zborowsky, Jemima Onime, Audrey Farbos, Paul O Neill, Aaron Jeffries, Christopher Jenkins, Isobel Norville, and Stefano Pagliara

Corresponding Author(s): Stefano Pagliara, University of Exeter

Review Timeline:

Submission Date:	March 13, 2026
Editorial Decision:	March 27, 2026
Revision Received:	June 30, 2026
Accepted:	July 2, 2026

Editor: Paolo Visca

Reviewer(s): The reviewers have opted to remain anonymous.

Transaction Report:

(Note: With the exception of the correction of typographical or spelling errors that could be a source of ambiguity, letters and reports are not edited. The original formatting of letters and referee reports may not be reflected in this compilation.)

DOI: https://doi.org/10.1128/spectrum.00881-26

Re: Spectrum00881-26 (Yersinia pseudotuberculosis employs a multifaceted strategy to survive antimicrobials)

Dear Prof. Stefano Pagliara:

Thank you for the privilege of reviewing your work. Below you will find my comments, instructions from the Spectrum editorial office, and the reviewer comments.

Both reviewers raised concern about the methodology and definitions, and suggest that your conclusions should be corroborated by additional experimental work. I agree with the reviewer's comments and I would reconsider your manuscript after extensive revision, including additional experiments to overcome the limitations highlighted by both reviewers.

Please return the manuscript within 90 days; if you cannot complete the modification within this time period, please contact me. If you do not wish to modify the manuscript and prefer to submit it to another journal, notify me immediately so that the manuscript may be formally withdrawn from consideration by Spectrum.

Revision Guidelines
To submit your modified manuscript, log into the submission site at https://spectrum.msubmit.net/cgi-bin/main.plex. Go to Author Tasks and click the appropriate manuscript title to begin. The information you entered when you first submitted the paper will be displayed; update this as necessary. Note the following requirements:

• Upload point-by-point responses to the issues raised by the reviewers in a file named "Response to Reviewers," NOT in your cover letter.
• Upload a compare copy of the manuscript (without figures) as a "Marked-Up Manuscript" file.
• Upload a clean .DOC/.DOCX version of the revised manuscript and remove the previous version.
• Each figure must be uploaded as a separate, editable, high-resolution file (TIFF or EPS preferred), and any multipanel figures must be assembled into one file.
• Any supplemental material intended for posting by ASM should be uploaded with their legends separate from the main manuscript. You can combine all supplemental material into one file (preferred) or split it into a maximum of 10 files with all associated legends included.

For complete guidelines on revision requirements, see our Submission and Review Process webpage. Submission of a paper that does not conform to guidelines may delay acceptance of your manuscript.

Data availability: ASM policy requires that data be available to the public upon online posting of the article, so please verify all links to sequence records, if present, and make sure that each number retrieves the full record of the data. If a new accession number is not linked or a link is broken, provide Spectrum production staff with the correct URL for the record. If the accession numbers for new data are not publicly accessible before the expected online posting of the article, publication may be delayed; please contact production staff (Spectrum@asmusa.org) immediately with the expected release date.

Publication Fees: For information on publication fees and which article types are subject to charges, visit our website. If your manuscript is accepted for publication and any fees apply, you will be contacted separately about payment during the production process; please follow the instructions in that e-mail. Arrangements for payment must be made before your article is published.

ASM Membership: Corresponding authors may join or renew ASM membership to obtain discounts on publication fees. Need to upgrade your membership level? Please contact Customer Service at Service@asmusa.org.

The ASM Journals program strives for constant improvement in our submission and publication process. Please tell us how we can improve your experience by taking this quick Author Survey.

Thank you for submitting your paper to Spectrum.

Sincerely,
Paolo Visca
Editor
Microbiology Spectrum

Reviewer #1 (Public repository details (Required)):

The manuscript presents transcriptomic analysis. Raw RNA-seq data should be deposited in public repository and the ID of repository should be included in the text.

Reviewer #1 (Comments for the Author):

The study shows that Y. pseudotuberculosis is difficult to eradicate with clinically relevant antibiotics and hydrogen peroxide in vitro, and it documents both tolerance and persistence phenotypes with fluoroquinolones. The manuscript is technically solid, however some points need clarification.

Comments:
1. Only one strain used in the study - please indicate this limitation.
2. The authors state that MIC experiments were performed based on CLSI protocol. According to CLSI the correct medium for MIC determination in MHB, the authors used LB. Please explain.
3. Citations for the "Clinical and Laboratory Standards Institute (CLSI) as previously described (109, 110)." are incorrect. Reference have to be revised thoroughly.
4. The Eagle effect claim needs stronger support with mechanistic experiments or the authors should be more careful with their interpretation.
5. The manuscript state that "overexpression of catalases and peroxidases plays a role in survival" however, it is not directly proven in the manuscript. This should be rephrased more cautiously or supported by minimal experiments.
6. Since this is a single-strain in vitro study statements such as "Y. pseudotuberculosis is difficult to kill via either antibiotics or disinfectants" should be qualified as "under the in vitro conditions tested" to avoid over-generalization.
7. The distinction between "exponential" and "stationary" phase is methodologically unclear. In the Results, susceptibility is reported after 6 h or 17 h of culture at 37 C, and the time-kill experiments are interpreted as probing stationary-phase populations but the Methods describe diluting overnight cultures into fresh LB and, for "exponential phase," an additional 6 h at 20 C. Please clarify.
8. The accession number of the raw RNA-seq data should be provided in the text.

Reviewer #2 (Comments for the Author):

In the manuscript entitled "Yersinia pseudotuberculosis employs a multifaceted strategy to survive antimicrobials" (ID 00881-26), the authors investigate the effects of different antibiotics and hydrogen peroxide on the killing of Y. pseudotuberculosis. The study suggests that persisters and the Eagle effect contribute to survival during exposure to quinolones, tolerance contributes to survival during ceftriaxone treatment, and catalases are involved in survival under hydrogen peroxide exposure. Global transcriptomic analysis (RNA-seq) was performed to clarify the transcriptional reprogramming that Y. pseudotuberculosis cells undergo during exposure to hydrogen peroxide.
The manuscript is generally understandable, although a careful language revision (e.g., using Grammarly or a similar editing tool) would further improve clarity and readability. The background is appropriate, and the relevant literature is generally well cited. Nevertheless, in its current form, the manuscript would benefit from substantial revision. A primary concern relates to the RNA-seq analysis, which is not accompanied by experimental validation. As presented, the transcriptomic data remain largely descriptive and appear somewhat disconnected from the initial phenotypic characterization following antibiotic treatment. The inclusion of targeted experiments to validate key differentially expressed genes, as well as to establish functional links with the observed phenotypes, would considerably enhance the biological relevance of the findings.
Overall, while the study addresses an interesting topic and contains potentially valuable observations, additional clarification and experimental support are needed before the manuscript can be considered for publication.
Major comments
1) According to Reference 13 cited by the authors (Brauner et al., 2016), MDK99 is defined as the minimum duration of treatment required to kill 99% of the initial inoculum (corresponding to a 2 $\log_{10}$ reduction). However, in lines 618-619, the authors state: "MDK99 [...] was defined as the minimum duration of treatment that led to a 3 $\log_{10}$ (99.9%) reduction in surviving bacteria compared to the initial inoculum." Therefore, the definition used in the manuscript more appropriately corresponds to MDK99.9, which is not consistent with the literature definition of MDK99. In fact, according to Reference 13, the MDK99 (2 $\log_{10}$ reduction) and MDK99.99 (4 $\log_{10}$ reduction) parameters are used to discriminate tolerant from persistent strains, whereas MDK99.9 is not defined in that framework. The authors should clarify and revise this definition accordingly.
2) In Figures 1, 2, and 3, the minimum limit of detection should be indicated.
3) In lines 279-281, the authors stated "Y. pseudotuberculosis was fully killed only after exposure to hydrogen peroxide used at 400×MIC value (i.e., 1M) with a MDK99 value of less than 1 h and no detectable persisters or regrowth after 24 h (Figure 3A)". However, in Figure 3A a small fraction of approximately 10 cells still appears to be detectable. Is 10 CFU/mL the limit of detection of this assay? The authors should clarify this point.
4) The gene clustering approach performed by the authors is not clearly explained, and it is unclear to this reviewer why a more conventional COG and/or KEGG-based functional classification was not used to group genes into established functional categories. As a consequence, Figure 4E-T is poorly informative in its current form, since many genes assigned to different clusters appear to belong to multiple functional categories. This makes it difficult to clearly interpret the metabolic and transcriptomic reprogramming that Y. pseudotuberculosis undergoes under the tested conditions. The authors should clarify the clustering strategy and consider complementing the analysis with a standard COG/KEGG functional enrichment to improve interpretability.
5) It is not entirely clear to this reviewer why hydrogen peroxide was selected as a major focus of the study, rather than

concentrating more extensively on antibiotics of clear clinical relevance. While hydrogen peroxide may induce oxidative stress, the rationale for its inclusion and its relationship to the antibiotic survival mechanisms investigated in the manuscript should be better justified and clarified.

6) The RNA-seq analysis is not followed by any experimental validation aimed at functionally linking the observed phenotype with the upregulation or downregulation of the most significantly differentially expressed genes (DEGs). As a result, the transcriptomic analysis appears largely descriptive and somewhat disconnected from the phenotypic observations presented in the study. Including targeted experiments to validate and functionally investigate some of the key DEGs would substantially strengthen the manuscript and help demonstrate the biological relevance of the transcriptomic findings.

Minor comments

1) The current title appears more suitable for a review article than for an original research paper. I recommend revising it to better reflect the specific focus of the study. For example: "Stress-induced survival of Yersinia pseudotuberculosis under clinical antimicrobials: contributions of persisters and the Eagle effect".

2) The Importance section should be revised. In its current form, it emphasizes that the MIC is used to define resistance but suggests that it is not useful for investigating bacterial killing. However, this interpretation is somewhat misleading. By definition, the MIC assay determines the minimum concentration required to inhibit bacterial growth, as its name indicates. When the objective is to assess bactericidal activity, the appropriate measure is the minimum bactericidal concentration (MBC) assay. This distinction does not imply that MIC is inadequate for measuring resistance levels, but rather that MIC and MBC address different aspects of antimicrobial activity. Instead of focusing on MIC, I suggest emphasizing and clarifying the definitions of persisters and the Eagle effect, as these concepts may be less familiar to the broader readership of Microbiology Spectrum. Providing a concise explanation would improve accessibility and context for non-clinical readers.

3) Since both tolerance and persisters are discussed in the Introduction, I suggest including their precise definitions as provided in Reference 13 of the manuscript (Brauner et al., 2016). This would ensure consistency with established terminology and improve clarity for readers.

4) Line 53 (Introduction): I recommend removing the word "often" from the sentence "Antibiotic resistance often occurs at the genotypic level". By definition, antibiotic resistance is a genetically encoded trait that is stable and heritable (see Reference 13 in the manuscript; Brauner et al., 2016).

5) In Table 1, please specify both measurement units for MIC and MBC in the corresponding columns, using the format "mg/L / mM".

6) Line 537: add "response" after "oxidative stress".

In the manuscript entitled "*Yersinia pseudotuberculosis* employs a multifaceted strategy to survive antimicrobials" (ID 00881-26), the authors investigate the effects of different antibiotics and hydrogen peroxide on the killing of *Y. pseudotuberculosis*. The study suggests that persisters and the Eagle effect contribute to survival during exposure to quinolones, tolerance contributes to survival during ceftriaxone treatment, and catalases are involved in survival under hydrogen peroxide exposure. Global transcriptomic analysis (RNA-seq) was performed to clarify the transcriptional reprogramming that *Y. pseudotuberculosis* cells undergo during exposure to hydrogen peroxide.

The manuscript is generally understandable, although a careful language revision (e.g., using Grammarly or a similar editing tool) would further improve clarity and readability. The background is appropriate, and the relevant literature is generally well cited. Nevertheless, in its current form, the manuscript would benefit from substantial revision. A primary concern relates to the RNA-seq analysis, which is not accompanied by experimental validation. As presented, the transcriptomic data remain largely descriptive and appear somewhat disconnected from the initial phenotypic characterization following antibiotic treatment. The inclusion of targeted experiments to validate key differentially expressed genes, as well as to establish functional links with the observed phenotypes, would considerably enhance the biological relevance of the findings.

Overall, while the study addresses an interesting topic and contains potentially valuable observations, additional clarification and experimental support are needed before the manuscript can be considered for publication.

Major comments

1) According to Reference 13 cited by the authors (Brauner *et al*., 2016), MDK99 is defined as the minimum duration of treatment required to kill 99% of the initial inoculum (corresponding to a 2 $\log_{10}$ reduction). However, in lines 618–619, the authors state: "MDK99 […] was defined as the minimum duration of treatment that led to a 3 $\log_{10}$ (99.9%) reduction in surviving bacteria compared to the initial inoculum." Therefore, the definition used in the manuscript more appropriately corresponds to MDK99.9, which is not consistent with the literature definition of MDK99. In fact, according to Reference 13, the MDK99 (2 $\log_{10}$ reduction) and MDK99.99 (4 $\log_{10}$ reduction) parameters are used to discriminate tolerant from persistent strains, whereas MDK99.9 is not defined in that framework. The authors should clarify and revise this definition accordingly.
2) In Figures 1, 2, and 3, the minimum limit of detection should be indicated.
3) In lines 279-281, the authors stated "*Y. pseudotuberculosis* was fully killed only after exposure to hydrogen peroxide used at 400×MIC value (*i.e*., 1M) with a MDK99 value of less than 1 h and no detectable persisters or regrowth after 24 h (Figure 3A)". However, in Figure 3A a small fraction of approximately 10 cells still appears to be detectable. Is 10 CFU/mL the limit of detection of this assay? The authors should clarify this point.
4) The gene clustering approach performed by the authors is not clearly explained, and it is unclear to this reviewer why a more conventional COG and/or KEGG-based functional classification was not used to group genes into established functional categories. As a consequence, Figure 4E–T is poorly informative in its current form, since many genes assigned to different clusters appear to belong to multiple functional categories. This makes it difficult to clearly interpret the metabolic and transcriptomic reprogramming that *Y. pseudotuberculosis* undergoes under the tested conditions. The authors should clarify the clustering strategy and consider complementing the analysis with a standard COG/KEGG functional enrichment to improve interpretability.
5) It is not entirely clear to this reviewer why hydrogen peroxide was selected as a major focus of the study, rather than concentrating more extensively on antibiotics of clear clinical relevance. While hydrogen peroxide may induce oxidative stress, the rationale for its

inclusion and its relationship to the antibiotic survival mechanisms investigated in the manuscript should be better justified and clarified.

6) The RNA-seq analysis is not followed by any experimental validation aimed at functionally linking the observed phenotype with the upregulation or downregulation of the most significantly differentially expressed genes (DEGs). As a result, the transcriptomic analysis appears largely descriptive and somewhat disconnected from the phenotypic observations presented in the study. Including targeted experiments to validate and functionally investigate some of the key DEGs would substantially strengthen the manuscript and help demonstrate the biological relevance of the transcriptomic findings.

Minor comments

1) The current title appears more suitable for a review article than for an original research paper. I recommend revising it to better reflect the specific focus of the study. For example: *"*Stress-induced survival of *Yersinia pseudotuberculosis* under clinical antimicrobials: contributions of persisters and the Eagle effect".
2) The Importance section should be revised. In its current form, it emphasizes that the MIC is used to define resistance but suggests that it is not useful for investigating bacterial killing. However, this interpretation is somewhat misleading. By definition, the MIC assay determines the minimum concentration required to inhibit bacterial growth, as its name indicates. When the objective is to assess bactericidal activity, the appropriate measure is the minimum bactericidal concentration (MBC) assay. This distinction does not imply that MIC is inadequate for measuring resistance levels, but rather that MIC and MBC address different aspects of antimicrobial activity. Instead of focusing on MIC, I suggest emphasizing and clarifying the definitions of persisters and the Eagle effect, as these concepts may be less familiar to the broader readership of *Microbiology Spectrum*. Providing a concise explanation would improve accessibility and context for non-clinical readers.
3) Since both tolerance and persisters are discussed in the Introduction, I suggest including their precise definitions as provided in Reference 13 of the manuscript (Brauner *et al*., 2016). This would ensure consistency with established terminology and improve clarity for readers.
4) Line 53 (Introduction): I recommend removing the word "often" from the sentence *"Antibiotic resistance often occurs at the genotypic level"*. By definition, antibiotic resistance is a genetically encoded trait that is stable and heritable (see Reference 13 in the manuscript; Brauner *et al*., 2016).
5) In Table 1, please specify both measurement units for MIC and MBC in the corresponding columns, using the format "mg/L / mM".
6) Line 537: add "response" after "oxidative stress".

Reviewer #1

The manuscript presents transcriptomic analysis. Raw RNA-seq data should be deposited in public repository and the ID of repository should be included in the text.

We have now deposited RNA-seq data in the NCBI Sequence Read Archive under BioProject accession PRJNA1480040. Associated SRA run accessions are SRR39232933-SRR39232951.

This information has been added to page 20, lines 667-671.

1) *Only one strain used in the study - please indicate this limitation.*

We have now acknowledged this limitation on page 4, lines 134-136, page 12, lines 380-381.

2) *The authors state that MIC experiments were performed based on CLSI protocol. According to CLSI the correct medium for MIC determination in MHB, the authors used LB. Please explain.*

Our initial rationale for using LB medium for MIC measurements was that this medium is typically used for time-kill assays for enumerating persisters and we wanted to use the same medium for MIC, MBC and time-kill assays for consistency.
However, following the reviewer comment, we have now measured the MIC values for the antibiotics tested in this study using cation-adjusted Muller-Hinton broth. We have found the same MIC values as previously tested in LB medium.

This information has been reported on page 16, lines 518-536.

3) *Citations for the "Clinical and Laboratory Standards Institute (CLSI) as previously described (109, 110)." are incorrect. Reference have to be revised thoroughly.*

We have now removed reference to guidelines from the Clinical and Laboratory Standards Institute (CLSI). We have referenced two previous works where we have provided extensive details on the protocols employed to carry out MIC measurements.

This point has been clarified on page 16, lines 514-515.

4) *The Eagle effect claim needs stronger support with mechanistic experiments or the authors should be more careful with their interpretation.*

We have now toned down our interpretation on how our data contribute to the understanding of the Eagle effect. Specifically, we have now stated "These paradoxical observations resemble the Eagle effect" on page 12, lines 406-407, and that "here we report for the first time that the efficacy of levofloxacin and ciprofloxacin against a *Y. pseudotuberculosis* strain decreases with the concentration of these drugs" on page 13, lines 417-419. We have also removed reference to the Eagle effect from the concluding paragraph on page 15.

5) *The manuscript state that "overexpression of catalases and peroxidases plays a role in survival" however, it is not directly proven in the manuscript. This should be rephrased more cautiously or supported by minimal experiments.*

Following the reviewers' comments, we have now carried out new experiments using quantitative real-time PCR to measure differential expression of 18 selected genes, including the genes encoding the catalase-peroxidase and the catalase enzymes KatG and KatA, of *Y. pseudotuberculosis* in response to 6 h exposure to hydrogen peroxide at a concentration of 62.5 mM. These measurements (performed on RNA extracted from triplicate control and exposed cultures, please see new Methods section) independently confirmed that *katG* and *katA* are strongly upregulated in *Y. pseudotuberculosis* in response to hydrogen peroxide, alongside *cybB* and *cybC*, encoding the cytochromes b561 and b562, the reductase *trxC*, the isocitrate lyase *aceA*, the malate synthase *aceB* and the isocitrate dehydrogenase *aceK*. We also confirmed that *Y. pseudotuberculosis* strongly downregulated the genes encoding the cytochrome ubiquinol oxidase subunits I and II CydA and CydB, the nitrate reductase complex NapAB, the chaperone NapD, the ferredoxin-type protein NapF, the nitrite reductase subunit NirB and NirD. Overall, we found a strong correlation between our genome-wide RNA sequencing data and our new qRT-PCR data (obtained using new experiments/cultures) with a Pearson correlation coefficient of 0.94 and a p-value < 0.0001.

These new data are presented in Figure 4H and on page 12, lines 352-356, with corresponding methods described on pages 18, lines 603-639.

We have also rephrased the sentence mentioned by the reviewer as following: "overexpression of catalases and peroxidases contributing to survival to hydrogen peroxide". This change has been implemented on page 1, lines 27-28.

6) *Since this is a single-strain in vitro study statements such as "Y. pseudotuberculosis is difficult to kill via either antibiotics or disinfectants" should be qualified as "under the in vitro conditions tested" to avoid over-generalization.*

We have now rephrased this statement as following "Under the *in vitro* conditions and strain tested in this study, *Y. pseudotuberculosis* is difficult to kill via either antibiotics or hydrogen peroxide" on page 4, lines 134-136.

7) *The distinction between "exponential" and "stationary" phase is methodologically unclear. In the Results, susceptibility is reported after 6 h or 17 h of culture at 37 C, and the time-kill experiments are interpreted as probing stationary-phase populations but the Methods describe diluting overnight cultures into fresh LB and, for "exponential phase," an additional 6 h at 20 C. Please clarify.*

We would like to clarify our approach here. Firstly, we carried out a growth curve experiment in triplicate, by picking three single colonies from a streak plate and by growing them in three separate flasks each containing 200 mL of LB medium incubated for 20 hours at 37 °C with shaking at 200 rpm. Aliquots were withdrawn from each culture at hourly time points to perform CFU assays that allowed to generate the data presented in Supplementary Figure 1.

Based on this data, we chose to perform MIC measurements for antibiotics against *Y. pseudotuberculosis* at two different time points: after 6 h and 17 h of growth, when *Y. pseudotuberculosis* was in exponential and stationary phase, respectively. Therefore, aliquots were withdrawn from a *Y. pseudotuberculosis* culture that had grown for 6 h or 17 h diluted to an OD_{600} of 0.01 and incubated at 37 °C for 6 hours either in cation-adjusted Muller-Hinton broth or in LB medium before their addition to the 96-well plate containing antimicrobials at a

final bacterial OD_{600} of 0.01. This dilution step was carried out to obtain a starting inoculation at roughly 1×10^6 CFU/mL.

We have now corrected the typo in the Methods section, these measurements were carried out consistently at 37 °C. We have also removed the word "further" that might have generated confusion. This point has been clarified on page 16, line 518.

We have also clarified on page 16, line 523-524 that "The dilution step was carried out to obtain a starting inoculation at roughly 1×10^6 CFU/mL."

8) *The accession number of the raw RNA-seq data should be provided in the text.*

This information has been added to page 20, lines 667-671.

Reviewer #2

1) *According to Reference 13 cited by the authors (Brauner et al., 2016), MDK99 is defined as the minimum duration of treatment required to kill 99% of the initial inoculum (corresponding to a 2 log_{10} reduction). However, in lines 618-619, the authors state: "MDK99 [...] was defined as the minimum duration of treatment that led to a 3 log_{10} (99.9%) reduction in surviving bacteria compared to the initial inoculum." Therefore, the definition used in the manuscript more appropriately corresponds to MDK99.9, which is not consistent with the literature definition of MDK99. In fact, according to Reference 13, the MDK99 (2 log_{10} reduction) and MDK99.99 (4 log_{10} reduction) parameters are used to discriminate tolerant from persistent strains, whereas MDK99.9 is not defined in that framework. The authors should clarify and revise this definition accordingly.*

Following the reviewer suggestion, we have provided the MDK_{99} and $MDK_{99.99}$ values for all drugs at all concentration tested in the new Table 2, we have reported these values in the relevant sections of the manuscript and we have provided the definition of these quantities on page 17, lines 559-561 in accordance with the guidelines in Reference 13.

2) In Figures 1, 2, and 3, the minimum limit of detection should be indicated.

We have clarified that in our time-kill assay measurements the limit of detection was 10 CFU ml^{-1} since (besides serially diluted samples) we also spotted 100 µl of undiluted samples. For samples in which we did not find any CFUs, we assigned an indicative numerical value of 1, i.e. lower than the limit of detection, but still compatible with the log_{10} scale representation used in the graphs. This point has been clarified on page 17, lines 554-558.

We have added dotted lines to indicate this limit of detection in all graphs presented in Figures 1, 2 and 3.

3) In lines 279-281, the authors stated "Y. pseudotuberculosis was fully killed only after exposure to hydrogen peroxide used at 400Å~MIC value (i.e., 1M) with a MDK99 value of less than 1 h and no detectable persisters or regrowth after 24 h (Figure 3A)". However, in Figure 3A a small fraction of approximately 10 cells still appears to be detectable. Is 10 CFU/mL the limit of detection of this assay? The authors should clarify this point.

We have now clarified in the point above that in our time-kill assay measurements the limit of detection was 10 CFU ml^{-1} since (besides serially diluted samples) we also spotted 100 µl of undiluted samples. For samples in which we did not find any CFUs, such as after exposure to hydrogen peroxide used at 400× MIC value, we assigned an indicative numerical value of 1, i.e. lower than the limit of detection, but still compatible with the log_{10} scale representation used in the graphs.

4) The gene clustering approach performed by the authors is not clearly explained, and it is unclear to this reviewer why a more conventional COG and/or KEGG-based functional classification was not used to group genes into established functional categories. As a consequence, Figure 4E-T is poorly informative in its current form, since many genes assigned to different clusters appear to belong to multiple functional categories. This makes it difficult to clearly interpret the metabolic and transcriptomic reprogramming that Y. pseudotuberculosis undergoes under the tested conditions. The authors should clarify the clustering strategy and consider complementing the analysis with a standard COG/KEGG functional enrichment to improve interpretability.

Indeed, we had performed gene set enrichment analysis (GSEA) to group genes belonging to each cluster into established functional categories. The results of this analysis were presented in Supplementary files S3-S10 of the submitted manuscript and described in the manuscript section titled "Gene expression changes triggered by exposure to hydrogen peroxide". Following this reviewer's comment we have now performed GSEA on pair-wise comparisons (i.e. 6 h 2.5 mM H2O2 vs untreated, 6 h 62.5 mM H2O2 vs untreated, 24 h 2.5 mM H2O2 vs untreated, 24 h 62.5 mM H2O2 vs untreated) rather than on the previously identified clusters. We agree with the reviewer that this approach allows to more readily identify biological processes that are significantly enriched in each comparison. We have therefore produced a new Figure 4 reporting the results of this GSEA analysis and removed the previous clustering analysis.

We have also implemented these changes on page 10, lines 313-316 and 332-337, page 11, lines 346-350, and page 18, lines 592-600.

5) It is not entirely clear to this reviewer why hydrogen peroxide was selected as a major focus of the study, rather than concentrating more extensively on antibiotics of clear clinical relevance. While hydrogen peroxide may induce oxidative stress, the rationale for its inclusion and its relationship to the antibiotic survival mechanisms investigated in the manuscript should be better justified and clarified.

In this study we set out to study the susceptibility of *Y. pseudotuberculosis* to representative molecules of antibiotic classes and one disinfectant that are routinely employed for antibiotic therapy or decontamination purposes while also investigating persistence and tolerance that are typically less investigated especially in understudied bacteria such as *Y. pseudotuberculosis*. We think we have clearly explained this point in our introduction, however, we welcome feedback from the reviewer should they find this point unclear.

Perhaps the focus of this reviewer's comment was on the rationale on following up with RNA sequencing for better understanding the response of *Y. pseudotuberculosis* to hydrogen peroxide rather than the antibiotics tested. Our rationale here was that we found very interesting that *Y. pseudotuberculosis* could regrow to levels similar to the initial inoculum even after

exposure to hydrogen peroxide at a concentration of 25× its MIC value. It would have been interesting to carry out this analysis also for the antibiotics tested and compare the biological responses elicited by each antimicrobial; however, this analysis would have requested resources beyond the ones available in this project.

We have clarified this rationale on page 4, lines 130-134.

6) The RNA-seq analysis is not followed by any experimental validation aimed at functionally linking the observed phenotype with the upregulation or downregulation of the most significantly differentially expressed genes (DEGs). As a result, the transcriptomic analysis appears largely descriptive and somewhat disconnected from the phenotypic observations presented in the study. Including targeted experiments to validate and functionally investigate some of the key DEGs would substantially strengthen the manuscript and help demonstrate the biological relevance of the transcriptomic findings.

We have fully taken onboard this feedback and carried out new experiments to address this point, please see our response to comment 5 of reviewer 1.

Minor comments
1) The current title appears more suitable for a review article than for an original research paper. I recommend revising it to better reflect the specific focus of the study. For example: "Stress-induced survival of Yersinia pseudotuberculosis under clinical antimicrobials: contributions of persisters and the Eagle effect".

We have now revised our title as following "*Contributions of persisters and the Eagle effect to survival of Yersinia pseudotuberculosis under clinical antimicrobials*"

2) The Importance section should be revised. In its current form, it emphasizes that the MIC is used to define resistance but suggests that it is not useful for investigating bacterial killing. However, this interpretation is somewhat misleading. By definition, the MIC assay determines the minimum concentration required to inhibit bacterial growth, as its name indicates. When the objective is to assess bactericidal activity, the appropriate measure is the minimum bactericidal concentration (MBC) assay. This distinction does not imply that MIC is inadequate for measuring resistance levels, but rather that MIC and MBC address different aspects of antimicrobial activity. Instead of focusing on MIC, I suggest emphasizing and clarifying the definitions of persisters and the Eagle effect, as these concepts may be less familiar to the broader readership of Microbiology Spectrum. Providing a concise explanation would improve accessibility and context for non-clinical readers.

We have now rewritten the importance section following this reviewer's advice.

3) Since both tolerance and persisters are discussed in the Introduction, I suggest including their precise definitions as provided in Reference 13 of the manuscript (Brauner et al., 2016). This would ensure consistency with established terminology and improve clarity for readers.

We have now used tolerance and persistence definitions in line with reference 13. This information has been added to page 2, lines 64-68.

4) Line 53 (Introduction): I recommend removing the word "often" from the sentence "Antibiotic resistance often occurs at the genotypic level". By definition, antibiotic resistance

is a genetically encoded trait that is stable and heritable (see Reference 13 in the manuscript; Brauner et al., 2016).

We have revised this sentence accordingly.

5) In Table 1, please specify both measurement units for MIC and MBC in the corresponding columns, using the format "mg/L / mM".

We have revised Table 1 accordingly.

6) Line 537: add "response" after "oxidative stress".

We have revised this sentence accordingly.

Re: Spectrum00881-26R1 (**Contributions of persisters and the Eagle effect to survival of *Yersinia pseudotuberculosis* under clinical antimicrobials**)

Dear Prof. Stefano Pagliara:

Thank you for submitting the revised version of your manuscript (Spectrum00881-26R1). I appreciated your replies to the Reviewers and the substantial improvements to your manucript.

Tehrefore, your revised manuscript is now accepted, and I am forwarding it to the ASM production staff for publication. Your paper will first be checked to make sure all elements meet the technical requirements. ASM staff will contact you if anything needs to be revised before copyediting and production can begin. Otherwise, you will be notified when your proofs are ready to be viewed.

Data Availability: ASM policy requires that data be available to the public upon online posting of the article, so please verify all links to sequence records, if present, and make sure that each number retrieves the full record of the data. If a new accession number is not linked or a link is broken, provide production staff with the correct URL for the record. If the accession numbers for new data are not publicly accessible before the expected online posting of the article, publication may be delayed; please contact ASM production staff immediately with the expected release date.

Publication Fees: For information on publication fees and which article types have charges, please visit our website. We have partnered with Copyright Clearance Center (CCC) to collect author charges. If fees apply to your paper, you will receive a message from no-reply@copyright.com with further instructions. For questions related to paying charges through RightsLink, please contact CCC at ASM_Support@copyright.com or toll free at +1-877-622-5543. CCC makes every attempt to respond to all emails within 24 hours.

ASM Membership: Corresponding authors may join or renew ASM membership to obtain discounts on publication fees. Need to upgrade your membership level? Please contact Customer Service at Service@asmusa.org.

Embargo Policy: A press release may be issued as soon as the manuscript is posted on the Spectrum Latest Articles webpage. The corresponding author will receive an email with the subject line "ASM Journals Author Services Notification" when the article is available online.

The ASM Journals program strives for constant improvement in our submission and publication process. Please tell us how we can improve your experience by taking this quick Author Survey.

Thank you for submitting your paper to Spectrum.

Sincerely,
Paolo Visca
Editor
Microbiology Spectrum
