# Supplementary material for: Contributions of persisters and the Eagle effect to survival of Yersinia pseudotuberculosis under clinical antimicrobials
Source: Microbiol Spectr. 2026 Jul 23;14(9):e00881-26. doi: 10.1128/spectrum.00881-26 (PMC13532240; doi:10.1128/spectrum.00881-26)
Supplement: Supplementary file 2 [file spectrum.00881-26-s0001.docx]

**Supplementary Figure 1** Temporal dependence of bacteria counts for *Y. pseudotuberculosis* growing in LB medium at 37 °C. The data points and error bars were calculated as the mean and standard deviation of CFU measurements carried out in biological triplicate each consisting of technical triplicate.

**Supplementary Figure 2** Principal component analysis of replicate transcriptomes of Y. pseudotubercolosis after 6 h (open symbols) or 24 h (filled symbols) of incubation in LB (circles) or exposure to 2.5 mM (squares) or 62.5 mM hydrogen peroxide (diamonds). Each transcriptome was measured in biological triplicate.
